# Unique Origin of Eye Canal Combines Elements of Blood, Lymph Tube Development

**DOI:** 10.1371/journal.pbio.1001913

**Published:** 2014-07-22

**Authors:** Richard Robinson

**Affiliations:** Freelance Science Writer, Sherborn, Massachusetts, United States of America

**Figure 1:**
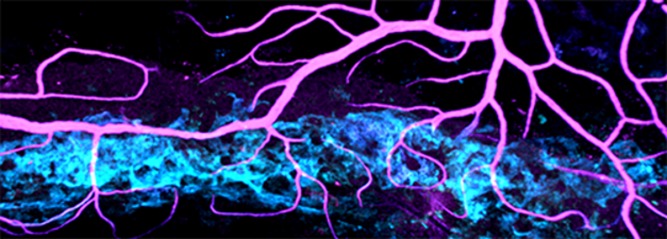
Schlemm's canal (cyan) is a key vessel that drains a clear fluid (aqueous humor) from the eye. The fluid drains into blood vessels (magenta) that are connected to the canal.

Sometimes things aren't either-or, but a little of both. That turns out to be the case for Schlemm's canal (Figure 1), a tiny but vital structure that drains fluid from the eye, keeping it healthy and preventing the build-up of pressure that can lead to glaucoma. Using an innovative combination of approaches, Krishnakumar Kizhatil, Simon John, and colleagues show that the development of Schlemm's canal is a unique process that shares features with the formation of both blood vessels and lymph vessels.

First, a bit of ocular anatomy. The eye's outer layer (the “ocular wall”) includes the sclera and, at the frontmost portion, the cornea. Behind the cornea is the fluid-filled anterior chamber. At the rear of the chamber sits the iris (with the lens just behind that). At its circumference, the iris meets the ocular wall. Just at this juncture, embedded within the ocular wall and encircling the edge of the disc-shaped anterior chamber, sits Schlemm's canal.

Schlemm's canal drains the fluid within the chamber, called aqueous humor, and regulates the passage of immune cells exiting the eye into the bloodstream. The details of the development of the canal have been controversial, in large part because it has been difficult to image the developing canal with sufficient clarity to follow the cellular fates of structures potentially involved.

The authors overcame this problem by developing a method for observing under the microscope the entire front portion of the eye, out to the border between the cornea and sclera (the limbus), including Schlemm's canal. Working in mice, this technique allowed them to study its development in unprecedented three-dimensional detail and to apply lineage-specific markers to determine the origin of cells making up the canal.

Prominent among their findings was that cells of the canal express not only characteristic blood vessel markers but also Prox1, a master regulator of lymphatic phenotypes. This suggested that canal cells might derive from lymphatic vessels, but by labeling and following cells expressing a second lymphatic marker, they ruled out a lymphatic origin for canal cells. Similarly, they were not derived from neural crest cells. Instead, the developmental program of canal cells appears to combine elements of angiogenesis, lymphangiogenesis, and vasculogenesis.

Detailed microscopic analysis indicated that canal cells derived from blood vessels of the limbus. Here, the authors found two vascular beds, oriented at right angles to each other, sandwiching the mature canal. During development, they found, the endothelial cells in each sprouted so-called tip cells, previously seen in development of new blood vessels from existing ones. In canal development, the authors observed tip cells growing out from their parent vessels, eventually clustering to form a continuous chain of cells around the limbus—the beginning of the canal. Over the ensuing days, Prox-1 expression increased, inner and outer walls of the canal began to develop, and finally, the lumen began to form.

The authors show that expression of KDR, a receptor for vascular endothelial growth factor, was essential to the process; however, most of the genetic and cell-signaling details governing this fascinating process remain to be worked out. Further work to understand canal formation will be greatly aided by the methods developed in this study. Understanding the details of canal development may lead to a better understanding of the working of the canal and how it can be manipulated to control glaucoma.

**Kizhatil K, Ryan M, Marchant JK, Henrich S, John SWM (2014) Schlemm's Canal Is a Unique Vessel with a Combination of Blood Vascular and Lymphatic Phenotypes that Forms by a Novel Developmental Process.** doi:10.1371/journal.pbio.1001912

